# Identification and comparative analysis of drought-associated microRNAs in two cowpea genotypes

**DOI:** 10.1186/1471-2229-11-127

**Published:** 2011-09-17

**Authors:** Blanca E Barrera-Figueroa, Lei Gao, Ndeye N Diop, Zhigang Wu, Jeffrey D Ehlers, Philip A Roberts, Timothy J Close, Jian-Kang Zhu, Renyi Liu

**Affiliations:** 1 Department of Botany and Plant Sciences, University of California, Riverside, CA 92521, USA; 2 Departamento de Biotecnologia, Universidad del Papaloapan, Tuxtepec Oaxaca 68301, Mexico; 3 Department of Horticulture and Landscape Architecture, Purdue University, West Lafayette, IN 47907, USA

## Abstract

**Background:**

Cowpea (*Vigna unguiculata*) is an important crop in arid and semi-arid regions and is a good model for studying drought tolerance. MicroRNAs (miRNAs) are known to play critical roles in plant stress responses, but drought-associated miRNAs have not been identified in cowpea. In addition, it is not understood how miRNAs might contribute to different capacities of drought tolerance in different cowpea genotypes.

**Results:**

We generated deep sequencing small RNA reads from two cowpea genotypes (CB46, drought-sensitive, and IT93K503-1, drought-tolerant) that grew under well-watered and drought stress conditions. We mapped small RNA reads to cowpea genomic sequences and identified 157 miRNA genes that belong to 89 families. Among 44 drought-associated miRNAs, 30 were upregulated in drought condition and 14 were downregulated. Although miRNA expression was in general consistent in two genotypes, we found that nine miRNAs were predominantly or exclusively expressed in one of the two genotypes and that 11 miRNAs were drought-regulated in only one genotype, but not the other.

**Conclusions:**

These results suggest that miRNAs may play important roles in drought tolerance in cowpea and may be a key factor in determining the level of drought tolerance in different cowpea genotypes.

## Background

Drought is one of the main abiotic factors that cause reduction or total loss of crop production. Because water is becoming limited for agriculture in many areas of the world, the investigation of natural mechanisms of drought tolerance is an important strategy for understanding the biological basis of response to drought stress and for selection of plants with improved drought tolerance [1,2]. Cowpea [*Vigna unguiculata *(L.) Walp.] is an economically important crop in semi-arid and arid tropical regions in Africa, Asia, and Central and South America, where cowpea is consumed as human food and nutritious fodder to livestock [3,4]. As a leguminous species, cowpea belongs to the same tribe (*Phaseoleae*) as common bean and soybean. Compared to these close relatives and most other crops, cowpea is well adapted to these regions because of its ability to fix nitrogen in poor soil and greater drought tolerance [4,5]. Therefore, cowpea is an excellent system for investigating the genetic basis of drought tolerance.

Efforts have been made to identify genetic elements that are involved in drought stress response in cowpea. For example, over a dozen genes have been shown to be associated with drought stress response through cloning and characterization of cDNAs [6-12]. In addition, ten drought tolerance quantitative trait loci (QTL) associated with tolerance in seedlings have been mapped in cowpea [13]. However, it is largely unknown how the expression of drought-associated cowpea genes or loci is regulated and how small RNAs are involved in the regulation.

MicroRNAs (miRNAs) are 20-24 nt single-stranded RNA molecules that are processed from RNA precursors that fold into stem-loop structures [14]. MiRNAs regulate gene expression of target mRNAs at the posttranscriptional level, which are recognized by nearly perfect base complementarity. Upon miRNA-target recognition, typically the target is negatively regulated via mRNA cleavage or translational repression [15]. Functional analyses have demonstrated that miRNAs are involved in a variety of developmental processes in plants [16]. In addition, miRNAs play critical roles in plant resistance to various abiotic and biotic stresses [17-19]. In particular, several approaches have been employed to study miRNAs that are involved in drought stress tolerance in plants. In one of the pioneering studies on stress-responsive miRNAs, Sunkar and Zhu [20] used small RNA cloning techniques to identify 26 novel miRNAs, among which miR393, miR397b, and miR402 were upregulated by dehydration and miR389a downregulated. Another miRNA family, miR169, was found to be downregulated by drought stress in an ABA-dependent pathway. The repression of miR169 leads to higher expression of its target gene NFYA5, which in turn enhances the drought resistance of the plant [21]. Many more miRNAs that are up- or down-regulated in drought condition were discovered by global miRNA expression profiling experiments with either microarray hybridization or small RNA deep sequencing [22-25].

Although numerous miRNAs have been identified in many plant species, including leguminous plants *Medicago truncatula *[26,27], soybean [28], and common bean [29], only two sequences have been reported for cowpea in the miRBase registry. Recently, 47 potential miRNAs belonging to 13 miRNA families were predicted in cowpea [30]. In another study, 18 conserved miRNAs belonging to 16 families were identified [31]. Both studies used a homology search approach to identify cowpea miRNAs that are conserved in other plants. In this study, we used Illumina deep sequencing technology to generate small RNA reads and used these reads to identify miRNAs in cowpea, especially cowpea-specific miRNAs and those associated with drought tolerance. To our knowledge, this is the first report of miRNAs identified through direct small RNA cloning in cowpea.

Despite inherent drought tolerance, cowpea varieties display significantly different levels of drought tolerance [32-34]. The study and comparison of plant genotypes differing in sensitivity to drought is a promising approach to discover natural tolerance mechanisms [35]. In order to gain insight into the role of miRNAs in tolerance to drought, we used two representative cowpea genotypes: California Blackeye No. 46 (CB46) and IT93K503-1. The drought-sensitive CB46 is the most widely grown blackeye-type cultivar in the United States and was developed at the University of California, Davis [36]. IT93K503-1 is a drought-tolerant breeding line developed by the International Institute of Tropical Agriculture (IITA) in Ibadan, Nigeria. We grew these two genotypes in well-watered and drought stress conditions and used leaves from the vegetative stage to construct four small RNA libraries. Using small RNA reads from these libraries, we identified 157 candidate miRNAs. Comparison of the expression pattern of miRNAs among libraries indicates that some miRNAs display different levels of expression in different genotypes, and thus may be a key factor to their different levels of drought tolerance.

## Results

### Identification of miRNAs in cowpea

**Figure 1:**
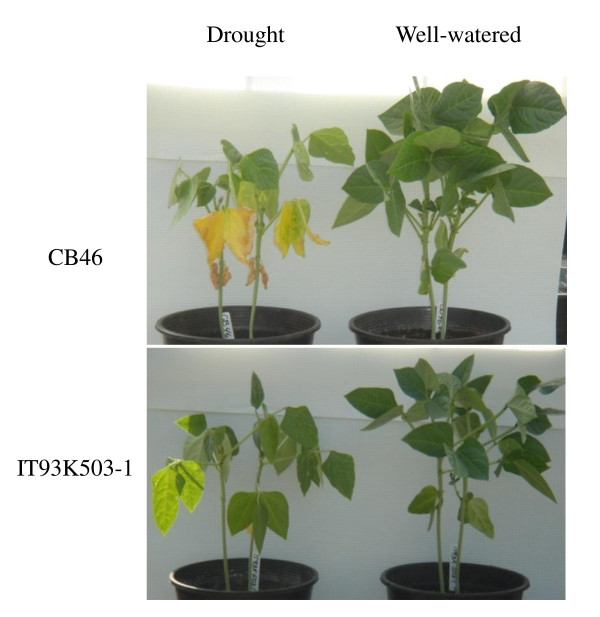
**Different drought tolerance of two cowpea genotypes**. After treated with moderate drought stress (*ψ_w _*= -1.5 MPa), IT93K503-1 plants continued to grow relatively well, but CB46 plants showed apparent symptoms of drought stress (chlorotic leaves).

In order to study the role of miRNAs in drought tolerance, we grew cowpea plants (CB46 and IT93K503-1) in green house under well-watered and drought stress conditions. Drought stress was applied to 30-day-old plants. After 10 to 15 days of moderate drought stress (*ψ_w _*= -1.5 MPa), the two genotypes showed apparent differences in drought tolerance. While IT93K503-1 plants continued to grow relatively well, CB46 plants displayed severe drought stress symptoms such as chlorotic leaves (Figure 1).

We constructed four small RNA libraries (2 genotypes × 2 growth conditions) and obtained on average 6.9 million (range: 6.5 - 7.3 million) clean small RNA reads from each library (deep-sequencing data have been deposited into the NCBI/GEO database with accession number GSE26402). The average number of unique reads per library is 4.3 million (range: 3.9 - 4.6 million). Using the procedure and criteria described in the materials and methods section, we mapped unique small RNA reads to a cowpea EST assembly, BAC end sequences and methylation filtration sequences, GSS sequences in dbGSS, and a draft cowpea genome assembly, and predicted 14, 78, 6, and 125 miRNA precursors, respectively. These four sets of putative miRNA precursors were then compared with each other to remove redundancy, and we obtained 157 candidate miRNA genes (for detailed information, see Additional file 1). Based on similarity of mature miRNA sequences, these miRNA genes were clustered into 89 families. Whereas 27 families (93 miRNAs) have match to miRNAs from other plants in the miRBase (release 16) [37], 62 families (64 miRNAs) appear to be cowpea-specific. Using a cowpea EST assembly, we have also identified putative target protein-coding genes for 112 (71%) miRNAs.

### Genotype-specific expression of miRNAs

**Figure 2:**
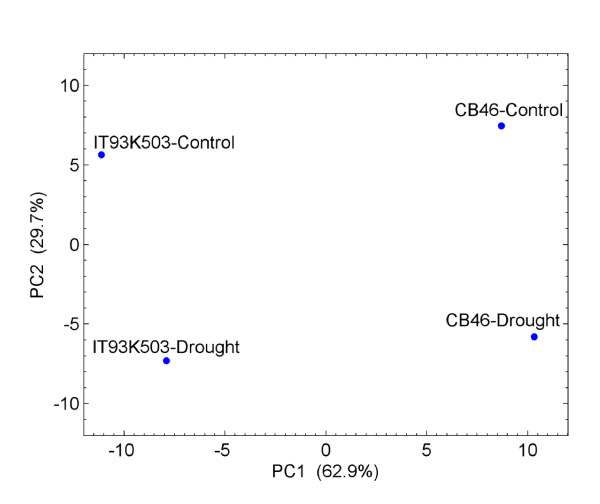
**Principal component analysis (PCA) of log2 miRNA normalized counts of two cowpea genotypes in two growth conditions**.

**Figure 3:**
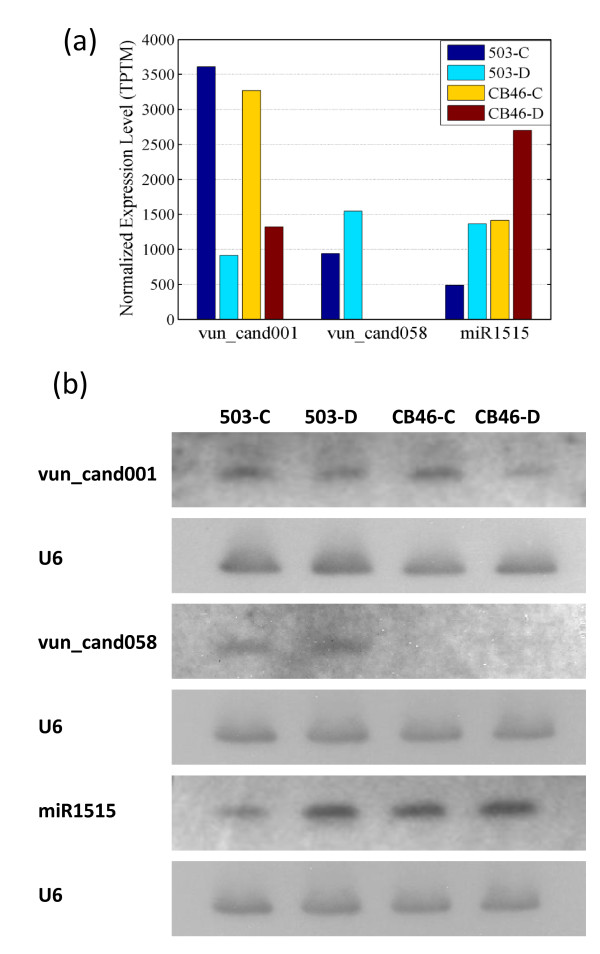
**Expression of selected miRNAs in two cowpea genotypes under two growth conditions**. Vun_cand001 and vun_cand058 are two cowpea-specific miRNAs, and miR1515 is a conserved miRNA that is also found in other plants. A. Expression level based on normalized miRNA counts (transcripts per ten million, TPTM). B. Northern blots with mature miRNAs. U6 snRNA was used to show equal loading of total RNA in all lanes.

Because small RNA libraries were sequenced to great depth, counts of mature miRNAs can be used to evaluate their relative expression levels in different genotypes and growth conditions. We first applied Principal Component Analysis (PCA) to the log2 normalized counts (transcripts per ten million, TPTM) of 91 unique mature miRNAs that had combined expression of at least 50 TPTM in four libraries. As shown in Figure 2, the first two components account for over 93% of variation in the data set, with the first component accounting for 63%. The first component (PC1) separates two samples of one genotype from two samples of the other genotype, indicating genotype is the main factor that determines miRNA expression levels. Indeed, nine miRNAs account for 75% of variation in PC1 and they show clear genotype-specific expressions (Table 1, for predicted hairpin structures and mapping of small RNA reads to precursors, see Additional files 2 and 3). Whereas two miRNAs (vun_cand014 and vun_cand054) are predominantly expressed in CB46, the other seven miRNAs are exclusively or predominantly expressed in IT93K503-1 plants. The expression pattern of IT93K503-1 specific miRNA, vun_cand058, was confirmed with northern blot assay (Figure 3b).

**Table 1:** MiRNAs that showed genotype-specific expression

		Normalized Expression Level (TPTM)*				
			
Family	Mature miRNA	IT93K503-Control	IT93K503-Drought	CB46-Control	CB46- Drought	Putative Target
vun_cand058	UUAAGCAGAAUGAUCAAAUUG	942	1546	3	0	hydroxyproline-rich glycoprotein
vun_cand048	UGGUCUCUAAACUUUAGAAAUGAA	746	263	0	2	
vun_cand036	UCAGAGGAAACAACACUUGUAC	59	23	0	0	
vun_cand045	CGUGCUGAGAAAGUUGCUUCU	52	79	14	5	VTC2 (vitamin c defective 2)
vun_cand053	GUAAUUGAGUUAAAAGGACUAUAU	43	6	0	2	cellulose synthase/transferase
vun_cand052	CGAGAGCCACUCGCCUAAGCGA	34	55	0	0	
vun_cand055	CCACUGUAGUAGCUCUCGCUCA	30	40	0	0	
vun_cand054	AGCAAGUUGAGGAUGGAGCUU	9	48	231	252	CKA1 (casein kinase alpha 1)
vun_cand014	UUCGGGAGUGAGAGCCAGUGA	3	0	56	5	UBP18 (ubiquitin-specific protease 18)

*TPTM: transcripts per ten million

Because perfect matches were required when small RNA reads were mapped to cowpea sequences for miRNA prediction, genotype-specific expression of miRNAs could be caused by inter-genotype single nucleotide polymorphisms (SNPs) in mature miRNAs. To address this possibility, we re-mapped clean small RNA reads from each library to the precursors of nine miRNAs in Table 1, allowing up to one mismatch. The normalized counts of these mature miRNAs were essentially unchanged (data not shown). Therefore, genotype-specific expression of these miRNAs was genuine and was not an artifact of the reads mapping process.

### Drought-associated miRNAs

To identify drought-associated miRNAs, we tested for differential expression of miRNAs in drought-stressed and corresponding control samples in each genotype using the statistical method developed by Audic and Claverie [38]. We used the following criteria to identify drought-associated miRNAs: (1) adjusted p-value was less than 0.01 in at least one of the two comparisons; (2) normalized counts (TPTM) was at least 100 in one of the four libraries; (3) log2 ratio of normalized counts between drought and control libraries was greater than 1 or less than -1 in one of the two genotypes. For differential expression analysis, we considered only unique mature miRNAs as they are the active form of the miRNA and in some cases, identical mature miRNA can be generated from two or more homologous miRNA genes. We found 44 drought-associated unique mature miRNAs that belong to 28 families (Additional file 4). The direction of statistically significant change was the same in both genotypes for all 44 miRNAs, indicating that miRNA gene expression in IT93K503-1 and CB46 had similar overall response to drought stress. Whereas thirty of 44 miRNAs were upregulated in the drought-stressed condition, fourteen were downregulated in one or both genotypes.

Among 44 drought-associated miRNAs, the expression of 22 miRNAs (17 families) in drought condition changed at least two-fold compared to the control in both genotypes (Additional file 4). Some of these miRNA families have been found to be associated with drought stress in previous studies, including miR156 and miR166 [39], miR159 [40], miR167 [24], miR169 [22], miR171 [25,41], miR395 [40], miR396 [24,39], and miR482 [29]. Most of the predicted targets encode transcription factors (Additional file 4). Other miRNA families, miR162, miR164, miR319, miR403, miR828, and four cowpea-specific miRNA (vun_cand001, vun_cand010, vun_cand041, and vun_cand057) were found to be associated with drought stress for the first time (northern blot confirmation of vun_cand001 was shown in Figure 3b).

We also found that 12 miRNAs showed at least two-fold change only in IT93K503-1 (Table 2), and 10 only in CB46 (Table 3). Although statistical tests indicated that some of these miRNAs (e.g. miR1515 which was validated by northern blot as shown in Figure 3b) were up- or down-regulated under drought stress in both genotypes without having two-fold change, 11 miRNAs were clearly regulated in only one genotype. Whereas miR160a, miR160b, miR171e, vun_cand015, vun_cand033, and vun_cand048 were significantly regulated by drought stress in IT93K503-1 plants only, miR171b, miR171d, miR2111b, miR390b, and miR393 were regulated only in CB46.

**Table 2:** MiRNAs that displayed at least two fold change under drought stress only in IT93K503-1

	Normalized Expression Level (TPTM)*								
miRNA ID	503-C	503-D	CB46-C	CB46-D	Log2(503-D/503-C)	Log2(CB46-D/CB46-C)	Adjusted p-value (503-D vs. 503-C)	Adjusted p-value (CB46-D vs. CB46-C)	Putative target
miR1515	489	1366	1415	2700	1.48	0.93	5e-63	4e-60	
miR160a	437	877	1048	1102	1.01	0.07	3e-21	1	ARF10
miR160b	13	244	139	178	4.18	0.35	9e-36	1	ARF10
miR167b	1649	4488	7539	13930	1.44	0.89	2e-201	1e-288	ARF8
miR171e	25	137	59	43	2.44	-0.45	4e-11	1	
miR319b	1019	2638	685	1215	1.37	0.83	5e-109	2e-21	transferase family protein
miR390a	2141	7242	3586	5308	1.76	0.57	0	3e-49	leucine-rich repeat transmembrane protein kinase
vun_cand009	582	1519	1025	1903	1.38	0.89	4e-63	4e-39	
vun_cand015	62	297	61	96	2.25	0.66	2e-23	1	RPL6A
vun_cand020	4462	12349	6115	10535	1.47	0.78	0	6e-177	pentatricopeptide repeat-containing protein
vun_cand033	478	172	96	113	-1.48	0.23	0	1	
vun_cand048	746	263	0	2	-1.51	N/A	0	1	

*TPTM: transcripts per ten million; 503-D and 503-C: IT93K503-1 under drought and control condition, respectively; CB46-D and CB46-C: CB46 under drought and control condition, respectively.

**Table 3:** MiRNAs that displayed at least two fold change under drought stress only in CB46

	Normalized Expression Level (TPTM)*								
					
miRNA ID	503-C	503-D	CB46-C	CB46-D	Log2(503-D/503-C)	Log2(CB46-D/CB46-C)	Adjusted p-value (503-D vs. 503-C)	Adjusted p-value (CB46-D vs. CB46-C)	Putative target
miR166a	7796	12734	7334	21341	0.71	1.54	4e-177	0	REV
miR171b	406	441	195	397	0.12	1.02	1	3e-09	mRNA guanylyltransferase
miR171d	58	55	85	215	-0.08	1.33	1	2e-07	
miR2111a	458	678	191	1105	0.57	2.53	5e-05	1e-106	kelch repeat-containing F-box protein
miR2111b	241	340	107	333	0.50	1.64	0.48	4e-17	kelch repeat-containing F-box protein
miR390b	52	33	110	34	-0.65	-1.69	1	8e-05	serine/threonine protein kinase
miR393	392	400	1120	258	0.03	-2.12	1	0	AFB3; AFB2
miR396b	4517	2958	5165	2411	-0.61	-1.10	0	0	growth-regulating factor 3
miR482	19518	10487	49339	13487	-0.90	-1.87	0	0	ARA12; serine-type endopeptidase
vun_cand030	848	431	531	196	-0.98	-1.44	0	0	zinc finger family protein

*TPTM: transcripts per ten million; 503-D and 503-C: IT93K503-1 under drought and control condition, respectively; CB46-D and CB46-C: CB46 under drought and control condition, respectively.

## Discussion

Regulation of gene expression through sequence-specific interaction between miRNAs and their target mRNAs offers an accurate and inheritable mechanism for plants to respond to environment stimuli [18]. Due to water limitations, drought is a major stress that limits the geographic distribution and yield of many crops. Therefore, extensive effort has been made for discovering genetic elements and mechanisms of drought tolerance, including the discovery of drought-associated miRNAs. As an important drought-tolerant crop in semi-arid and arid areas, cowpea offers a good system for the study of drought tolerance. Here we used deep sequencing of small RNA libraries from two cowpea genotypes and identified 157 miRNAs. By comparing the expression level of miRNAs in drought-stressed sample to control sample, we also identified 30 miRNAs that were upregulated in drought condition and 14 downregulated. This list of drought-associated miRNAs includes miRNA families that were known to be associated with drought in other plant species, indicating that they are involved in conserved drought response pathways. Some miRNA families, including some cowpea-specific miRNAs, were found to be associated with drought for the first time, suggesting that they may be involved in lineage- or species-specific stress response pathways and functions.

We predicted target genes for 32 out of 44 drought-associated miRNAs. The predicted target mRNAs encode proteins of diverse function, most of them being transcription factors (Additional file 4). For most of the conserved miRNAs, it is expected that their targets are also conserved. For example, our results showed that miR156 was upregulated in response to drought in cowpea. MiR156 has been known to be responsive to abiotic stresses and targets SPB transcription factors in Arabidopsis, maize, rice and wheat [24,39,41-43]. This miRNA is also involved in the regulation of development during vegetative phase change [42], indicating that reprogramming of development is a crucial step in plants to cope with drought stress. Another miRNA, miR169, was downregulated in both cowpea genotypes. In Arabidopsis, miR169 was downregulated and its target, a Nuclear Factor Y transcription factor NFYA5, was induced by drought stress [21]. MiR169 most likely functions in a similar way in cowpea to enhance drought tolerance by inducing the expression of NFYA5 orthologs.

The cowpea genotypes studied in this work have different abilities of drought tolerance. Because the two genotypes are highly similar to each other in their genetic composition, their phenotypic variations such as drought tolerance are most likely caused by changes in regulatory processes, rather than changes in proteins [44]. Due to their different geographical origins, the two genotypes are adapted to the particular environmental conditions in their natural habitats. It is thus expected to find constitutive differences, which could be related to metabolism, use of energetic resources, mobilization of biomass, structure of radical system, wax deposition in leaves, membrane stability or density of stomata, among other characteristics. We found that nine miRNAs were predominantly or exclusively expressed in only one genotype, regardless of the treatments. On the other hand, 11 miRNAs were found to be differentially expressed under drought stress in one genotype, but not the other. Changes in miRNA expression are expected to cause changes in the expression of target genes between the two genotypes.

Among miRNAs that had genotype-specific regulation, miR160a and miR160b were upregulated in response to drought in the tolerant, but not in the sensitive cultivar (Table 2). Their putative targets are members of the family of Auxin Response Factors (ARFs). ARFs are key elements in regulation of physiological and morphological mechanisms mediated by auxins that may contribute to stress adaptation [45]. Moreover, negative regulation of ARF10 by miR160 was demonstrated to be critical during seed germination in *Arabidopsis thaliana *through the crosstalk between auxin and ABA-dependent pathways [46]. On the other hand, two members of the miR2111 family were upregulated by drought in the sensitive, but not in the tolerant cultivar (Table 3). Their putative targets are Kelch repeat-containing F-box proteins that belong to a large family with members known to be involved in response to biotic and abiotic stresses [47]. Furthermore, F-box proteins containing Kelch repeats have been found to be responsive to drought in chickpea, a close relative of cowpea [48]. This suggests that genotype-specific regulation of miRNAs might be part of the reason why some cowpea genotypes have stronger drought tolerance than others.

Among the new miRNA candidates that were identified in this study, ten were regulated by drought stress and target genes were predicted for five of them. For instance, vun_cand030 was downregulated by drought and putatively targets a zinc finger protein. Zinc finger proteins are known to be involved in a variety of functions in development and stress response [49]. Moreover, vun_cand015 was upregulated by drought in the tolerant cultivar and putatively targets a basic-helix-loop-helix (bHLH) transcription factor. These proteins have roles in response to abiotic stresses, such as iron deficiency [50], freezing, and salt stress [51]. This suggests these new miRNAs may be indeed an integral component of drought response in cowpea.

For many miRNAs there were more than one target predicted. The possibility of a miRNA to have multiple targets is commonly observed. To confirm these predicted targets, we need to perform detailed analysis of cleavage of mRNA targets at the miRNA recognition site by experimental approaches, such as RACE and degradome analysis [52-54]. Once we validate the targets of drought-associated miRNAs, we will be in a better position to link the expression changes of miRNAs and their targets to differences of drought tolerance in cowpea.

Because we do not have the complete cowpea genome sequence, some miRNA genes were not identified, even though they had significant expression in our small RNA libraries. To find out how many miRNA families have been missed, we mapped unique small RNA reads to plant miRNA precursors in the miRBase, allowing up to 2 mismatches. Although we did not miss a large number of miRNAs, we did find that miR2118, miR2911, and miR529 had significant expression in our libraries (Additional file 5). The latter two were also induced by drought stress. MiR529 was identified as drought-associated miRNA in rice [25]. However, contrary to the pattern that we found in cowpea, it was downregulated under drought stress in rice. It is not clear whether it was caused by different sampling time or tissue, or species-specific stress response mechanisms.

Like protein coding genes, many miRNA families possess more than one miRNA gene and miRNA genes from the same family may have either identical or similar but different mature miRNA sequences. During evolutionary process, homologous miRNA genes may functionally diverge from each other. In the set of miRNAs that we identified in cowpea, members from miR166 and miR167 families showed clear evidence for functional diversification. While one member miRNA gene (miR166a, miR167b) was induced by drought stress, another miRNA from the same family (miR166b, miR167a) was significantly downregulated (Additional file 4).

## Conclusions

Using deep sequencing technology, we identified 157 miRNAs in cowpea, including 44 miRNAs that are drought-associated. By comparing mature miRNA counts in different genotypes and growth conditions, we found 9 miRNAs that were almost exclusively expressed in only one genotype and 11 miRNAs that were regulated by drought stress in one genotype, but not the other. Our study demonstrated that deep sequencing of small RNAs is a cost-effective way for miRNA discovery and expression analysis. Compared to the homology search method, deep sequencing allowed the detection of species-specific miRNAs and digital expression analysis. Our findings demonstrate that expression patterns of some miRNAs may be very different even between two genotypes of the same species. Further characterization of the targets of drought-associated miRNAs will help understand the details of response and tolerance to drought in cowpea.

## Methods

### Plant materials

CB46 and IT93K503-1 plants were grown in a greenhouse at the University of California Riverside campus in Spring 2009. The temperature was 35°C during the day and 18°C at night with no artificial control of day length. Four seeds were germinated in 2 gallon-pots filled with steam-sterilized UC Riverside soil mix UCMIX-3 and thinned to two plants per pot two weeks after planting. Three replicate pots per treatment were arranged in a completely randomized block design. When plants were 30 days old, corresponding to late vegetative stage, deficit irrigation treatments were applied by withholding watering on the stressed pots while controlled pots were water daily to soil capacity. Third leaf water potential was monitored using a pressure chamber (Cornallis, PMS instruments, USA) [55] as the indicator of the stress level. Fresh leaves (second from apex) of three replicates were sampled and frozen in liquid nitrogen from control plants (well watered, *ψ_w _*= -0.5 MPa) and moderately stressed plants (*ψ_w _*= -1.5 MPa) for RNA extraction.

### Small RNA library construction and sequencing

Total RNA was extracted with the TRIzol reagent (Invitrogen) according to the manufacturer's instructions. Small RNA libraries were constructed from cowpea leaves using the procedure used by Sunkar and Zhu [20] with minor modifications [56]. Briefly, for each treatment/genotype group, equal amount of total RNA was pooled from three replicates to generate ~700 μg of RNA. Pooled total RNA was resolved in a 15% denaturing polyacrylamide gel and the 20-30 nt small RNA fraction was extracted and eluted. A preadenylated adaptor (linker 1, IDT) was ligated to the 3' end of small RNAs with the use of T4 RNA ligase. Ligation products were then gel purified and subsequently ligated to an RNA adaptor at the 5'end. After ligation and purification, the products were used as template for RT-PCR. After synthesis and purification, the PCR products were quantified and sequenced using an Illumina Genome Analyzer.

### miRNA identification

Only small RNA reads that passed the Illumina quality control and contained clear adaptor sequences were considered good reads for further processing. After adaptor sequence was trimmed, clean small RNA reads of 18nt or more were combined into unique sequences. Reads that match known plant repeats, rRNAs, tRNAs, snRNAs, and snoRNAs were removed. Unique small RNA reads were mapped to four genomic sequence resources with SOAP2 [57]: cowpea EST assembly available in HarvEST:Cowpea [58] (http://harvest.ucr.edu, version 1.17, 18,745 sequences, but we excluded those appear to be protein-coding genes), a combination of 260,642 cowpea gene-space random shotgun sequences [59] and 30,527 BAC end sequences (obtained from M.-C. Luo, UC Davis, http://phymap.ucdavis.edu:8080/cowpea), 54,123 cowpea Genome Survey Sequences (GSS) from dbGSS of GenBank http://www.ncbi.nlm.nih.gov/dbGSS/, and a draft cowpea genome assembly from 63× coverage Illumina pair-ended reads (296,868 contigs with total length of ~186 MB, available at http://www.harvest-blast.org). Perfect match was required.

We used the updated annotation criteria for plant miRNAs [60] and built an in-house pipeline for miRNA prediction. Unique reads with a redundancy of at least 10 copies are used as anchor sequences. With one end anchored at 10 bp from the mapped position, DNA segments of 100 - 300 bp that cover each anchor sequence were sampled with 20 bp as step size. Secondary structure of each segment was predicted with UNAFold [61]. We then examined the structures and only those met the following criteria were considered genuine miRNA candidates: (1) free energy is lower than or equal to -35 kcal/mol; (2) number of mismatches between putative miRNA and miRNA* is 4 or less; (3) number of asymmetrical bulges in the stem region is not greater than 1 and the size of each asymmetrical bulge is 2 or less; (4) strand bias - small RNA reads that map to the positive strand of the hairpin DNA segment account for at least 80% of all mapped reads; (5) precise cleavage - reads that map to the miRNA and miRNA* regions (defined as miRNA or miRNA* plus 2nt on 5' and 3' ends) account for at least 75% of all reads that map to the precursor. If two or more candidate hairpins were predicted from the same region, we compared these hairpins and chose a hairpin that has highest putative mature miRNA expression, lowest free energy, or shortest length.

In order to classify miRNAs into families, all predicted mature miRNAs were compared with themselves using the ssearch35 program in the FASTA package (version 3.5) [62]. Using a single-linkage algorithm, mature miRNAs with up to two mismatches were included in same clusters. Mature miRNAs were then compared with the mature miRNAs in the miRBase (Release 16) [37] using ssearch35. If a member in a cowpea miRNA cluster had a match (allowing up to two mismatches) in the miRBase, the family number of the known miRNA was assigned to the cluster, otherwise the cluster was annotated as a new family.

### miRNA Target prediction

Mature miRNA sequences were used as query to search the cowpea EST assembly for potential target sites using miRanda [63]. The alignments between miRNAs and potential targets were extracted from the miRanda output and scored using a position-dependent, mispair penalty system [64-66]. Briefly, miRNA-target duplexes were divided into two regions: a core region that includes positions 2-13 from the 5' end of the miRNA, and a general region that contains other positions. In the general region, a penalty score of 1 was given to a mismatch or a single-nucleotide bulge or gap, and 0.5 to a G:U pair. Scores were doubled in the core region. A match was considered positive if the alignment between miRNA and target meets two conditions: (1) the penalty score is 4 or less; (2) total number of bulges and gaps is less than 2.

### Principal component analysis

Counts of each mature miRNA were first normalized to transcripts per ten million (TPTM) according to the total number of clean small RNA reads in each of the four libraries. MiRNAs with combined expression of at least 50 TPTM were chosen for principal component analysis (PCA). We used the log2 values of miRNA normalized counts to build an expression matrix and used the princomp function in MATLAB (MathWorks Inc., Natick, MA) for PCA.

### Statistical test for differential expression of miRNAs

Because deep sequencing of small RNAs provides a random sampling of mature miRNAs in the original small RNA pools, counts of miRNAs can be modeled by a Poisson distribution. We applied an established method [38,67] to calculate the p-value for differential expression of miRNAs between a drought-stressed sample and a control sample. The first step was to calculate a conditional probability using the formula:

$$p\left(y|x\right)={\left(\frac{N_{2}}{N_{1}}\right)}^{y}\frac{\left(x+y\right)!}{x!y!{\left(1+\frac{N_{2}}{N_{1}}\right)}^{\left(x+y+1\right)}}$$

Where *N_1 _*is total number of clean reads in the control library, *N_2 _*is total number of clean reads in the drought-stressed library, *x *is number of a mature miRNA in the control library, and *y *is number of the same mature miRNA in the drought-stressed library. A two-tailed p-value for differential expression was then calculated as *p *= 2*q*, where q was the accumulated probability:

$$q=\overset{y^{\prime }\leq y}{\underset{y^{\prime }=0}{\sum }}p\left(y^{\prime }|x\right)$$

Due to the *x*↔*y *symmetry of *p*(*y|x*), if *q *was greater than 0.5, p-value could be calculated as *p *= 2*(1-*q*). Bonferroni method was used to adjust p-values for multiple comparisons.

### Northern blot analysis

~40 μg of total RNA were resolved in 15% denaturing polyacrylamide gels and transferred to neutral nylon membranes (Hybond NX). The RNA was transferred and fixed to the membranes by chemical cross-linking [68] and then hybridized to probes complementary to mature miRNA sequences at 38°C, overnight. After hybridization, the blots were washed twice, 5 minutes each at 38°C with washing solution (2X SSC, 0.1% SDS) and exposed to X-ray film to reveal the signals. Results obtained in Northern blot assays were verified in three replicated samples.

## Authors' contributions

BEB-F, J-KZ, TJC and RL conceived the study. BEB-F, ZW, NND, JDE, and PAR carried out the experiments. BEB-F, LG, J-KZ, and RL analyzed the data, LG contributed new analysis tools, RL, BEB-F, TJC, and J-KZ wrote the paper. All authors read and approved the final manuscript.

## Supplementary Material

Additional file 1 **MiRNAs that were identified in cowpea**. Detailed information of the predicted cowpea miRNAs and their targets. Click here for file

Additional file 2 **Predicted hairpin structures of nine genotype-specific miRNAs**. Predicted structures of nine genotype-specific miRNAs with mature miRNAs marked in green. Click here for file

Additional file 3 **Mapping of small RNA reads from four libraries to the precursors of nine genotype-specific miRNAs**. Each figure shows the precursor sequence, predicted hairpin structure, and how each unique read was mapped to the precursor. Click here for file

Additional file 4 **Drought-associated miRNAs in cowpea**. Detailed information of drought-associated miRNAs and their targets. Click here for file

Additional file 5 **Other conserved miRNAs that were expressed in cowpea**. Three conserved miRNAs and their expression values in two cowpea genotypes under two growth conditions. Click here for file
