# Supplementary material for: Identification and comparative analysis of drought-associated microRNAs in two cowpea genotypes
Source: BMC Plant Biol. 2011 Sep 17;11:127. doi: 10.1186/1471-2229-11-127 (PMC3182138; doi:10.1186/1471-2229-11-127)
Supplement: Additional file 2 — Predicted hairpin structures of nine genotype-specific miRNAs. Predicted structures of nine genotype-specific miRNAs with mature miRNAs marked in green. [file 1471-2229-11-127-S2.PDF]

Predicted hairpin structures of nine genotype-specific miRNAs

Free energy and miRNA family name are on the bottom of each structure. Nucleotides that constitute mature RNAs are drawn in green.

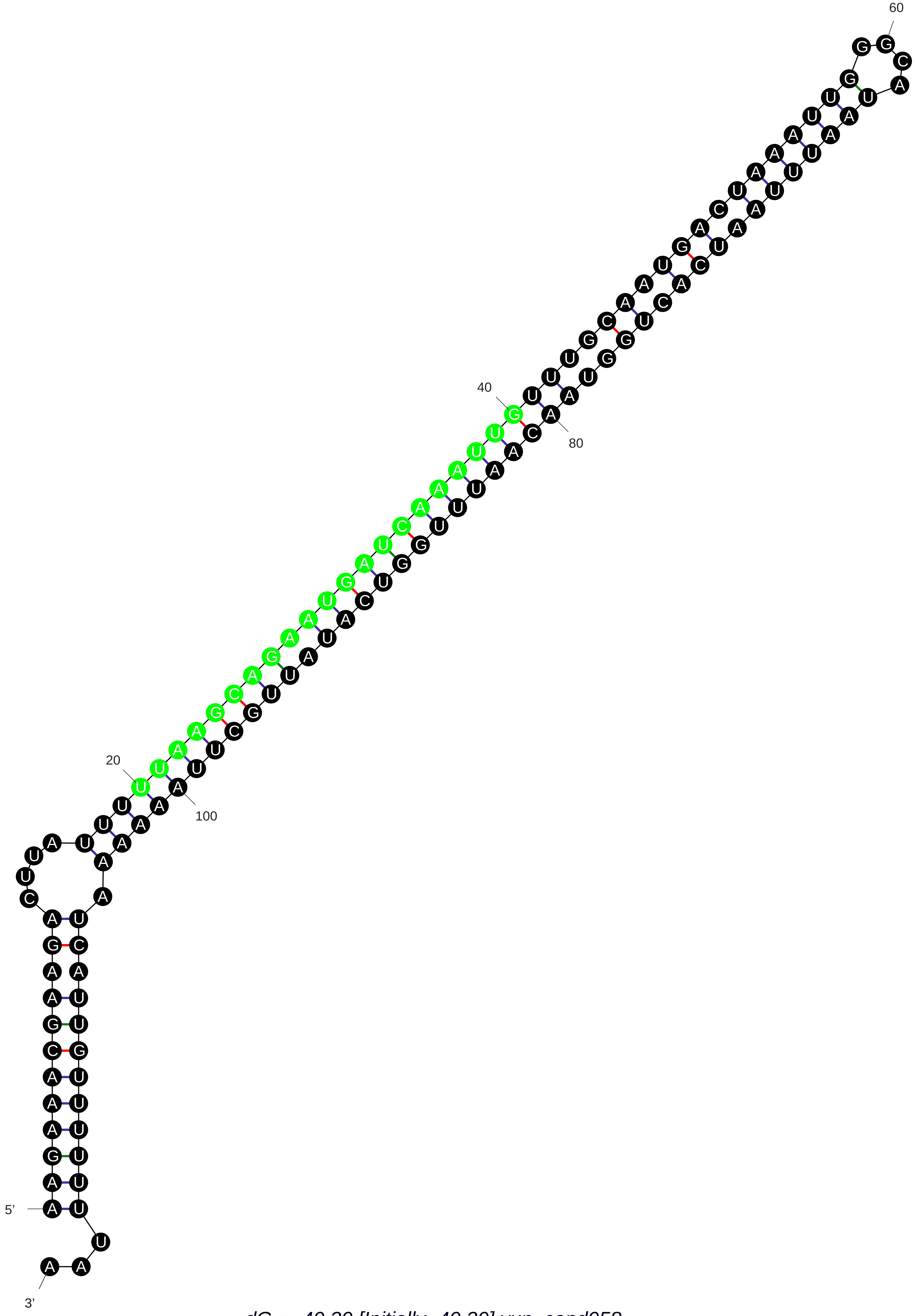

dG = -40.30 [Initially -40.30] vun_cand058

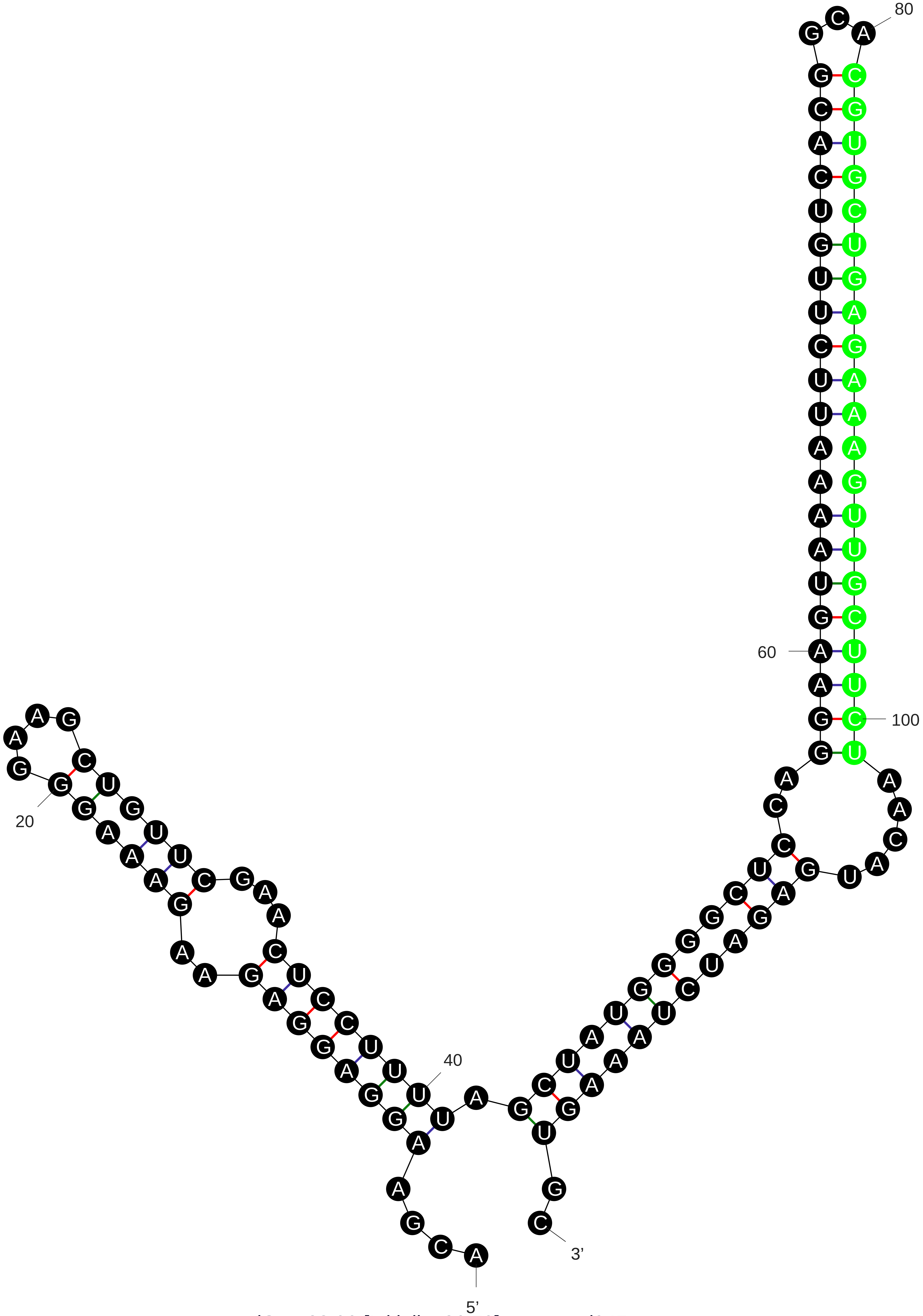

dG = -38.90 [Initially -36.50] vun_cand045

Output of sir_graph (©)
mfold_util 4.6

Created Wed May 11 01:27:09 2011

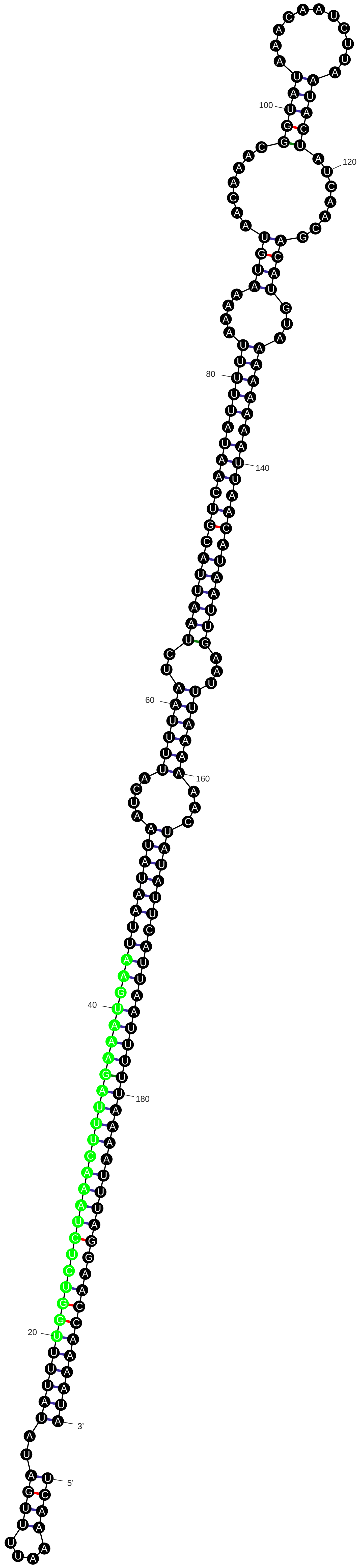

dG = -35.60 [Initially -35.60] vun_cand048

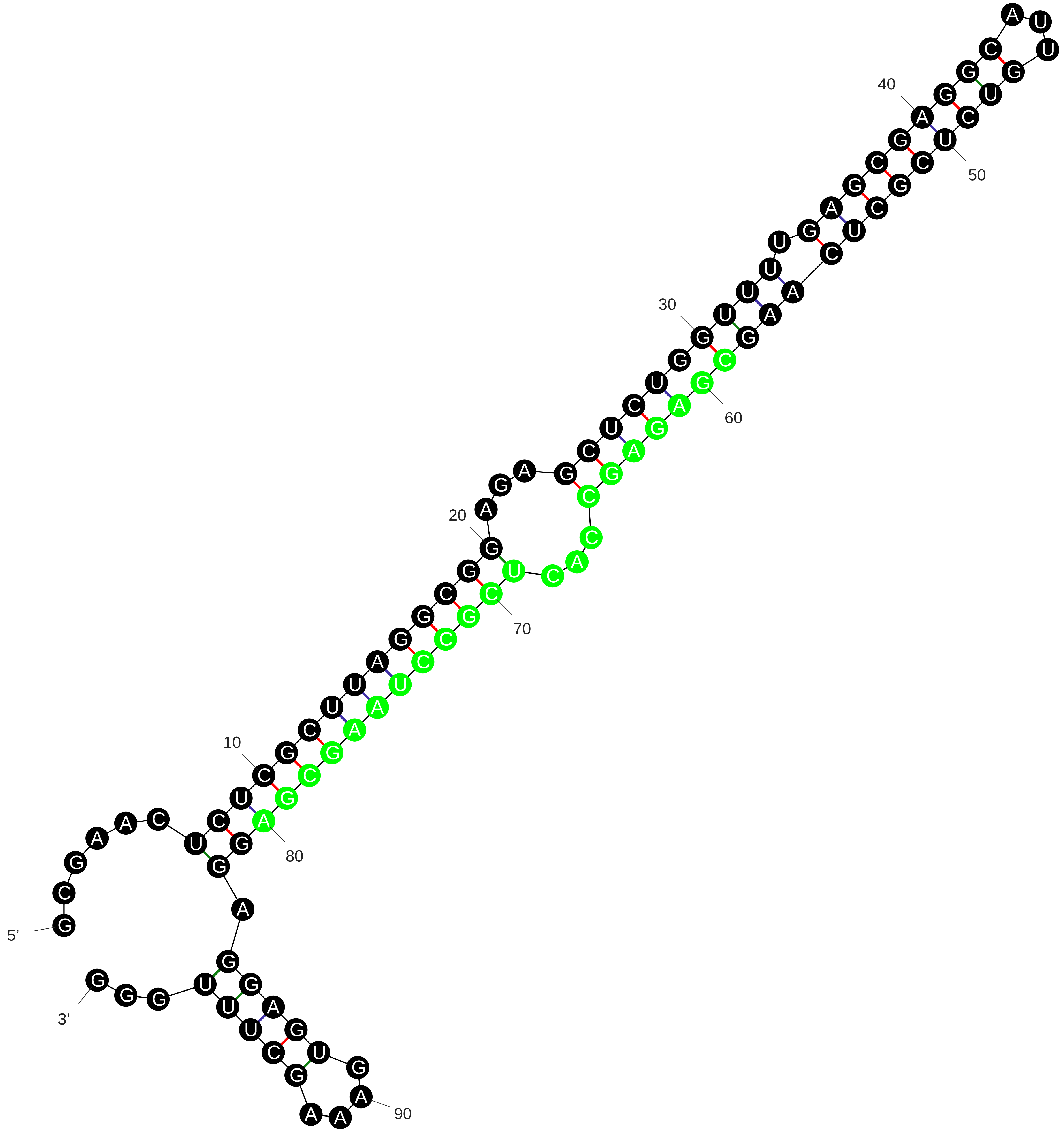

dG = -59.20 [Initially -56.70] vun_cand052

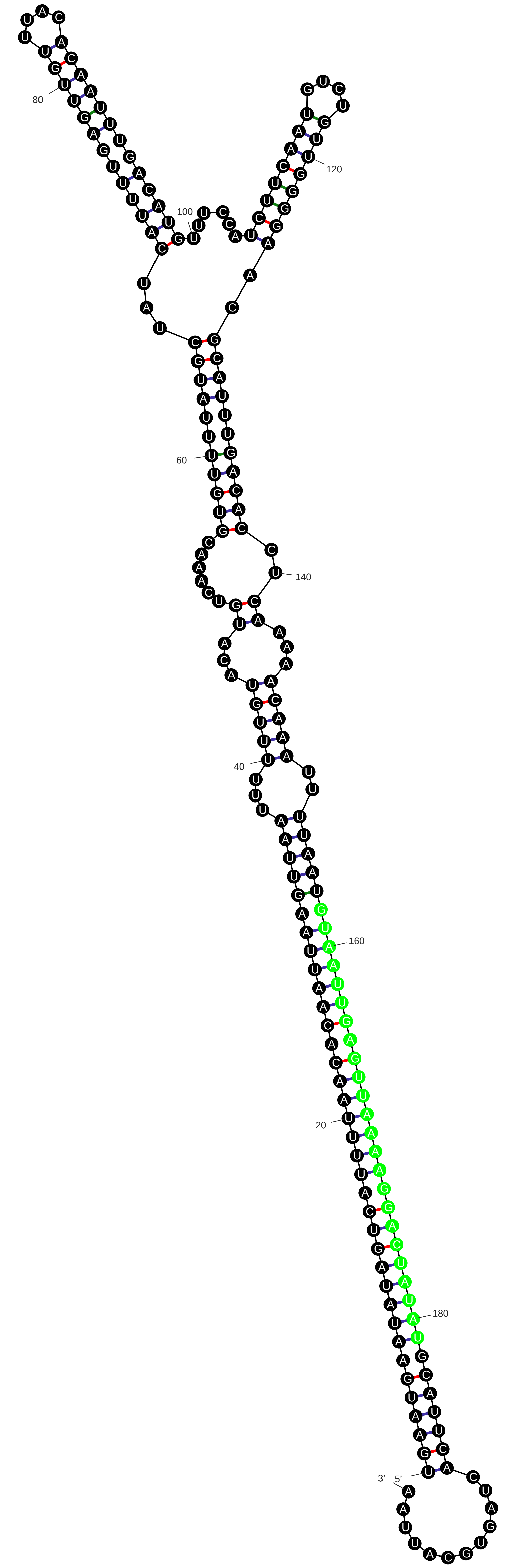

dG = -52.33 [Initially -56.20] vun_cand053

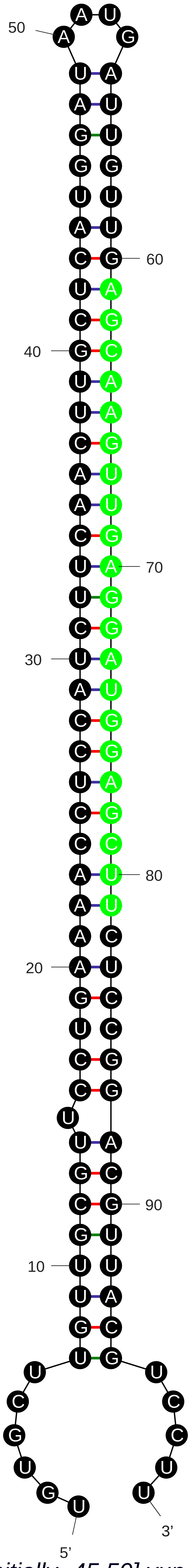

dG = -45.50 [Initially -45.50] vun_cand054

Output of sir_graph (©)
mfold_util 4.6

Created Wed May 11 01:37:36 2011

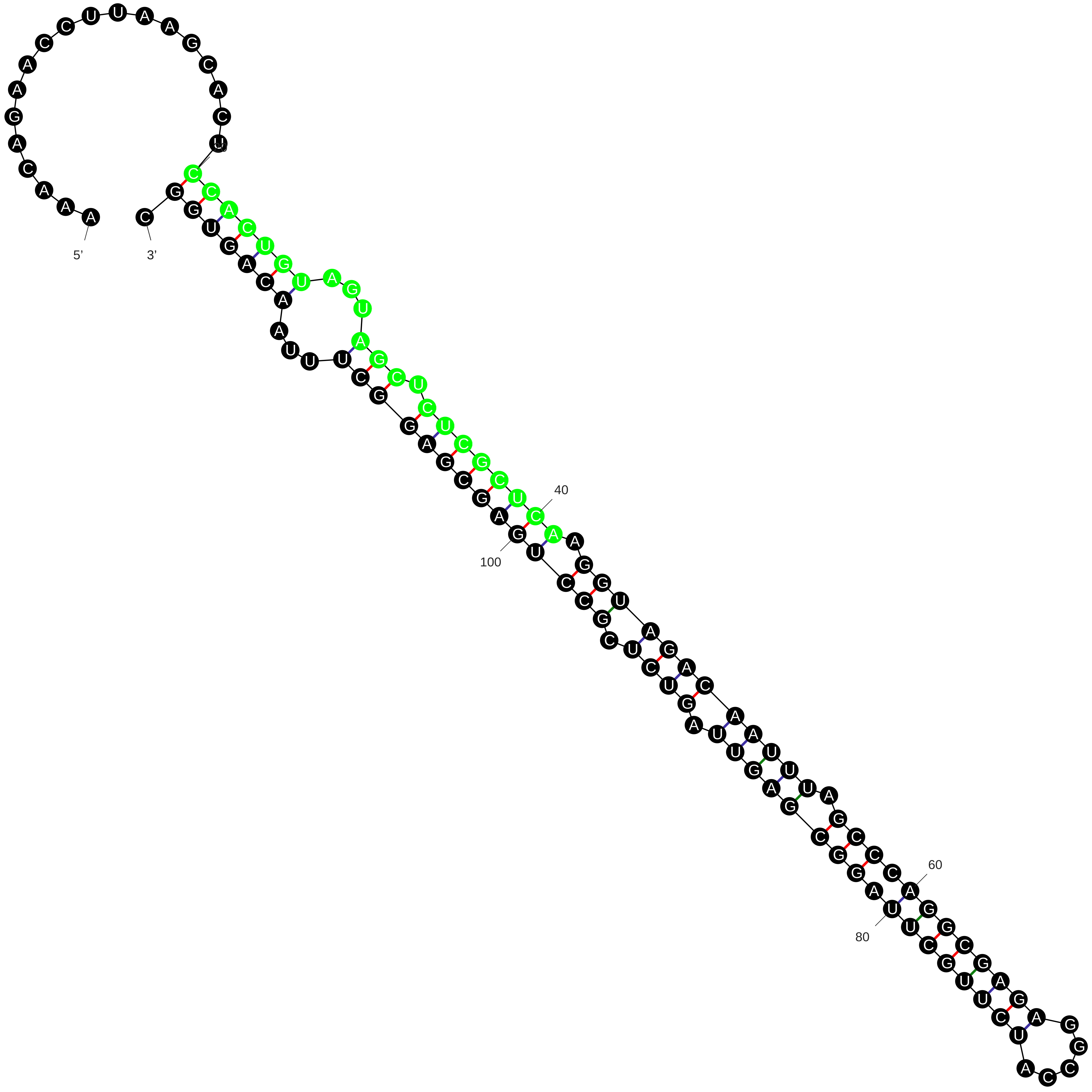

dG = -56.20 [Initially -56.20] vun_cand055

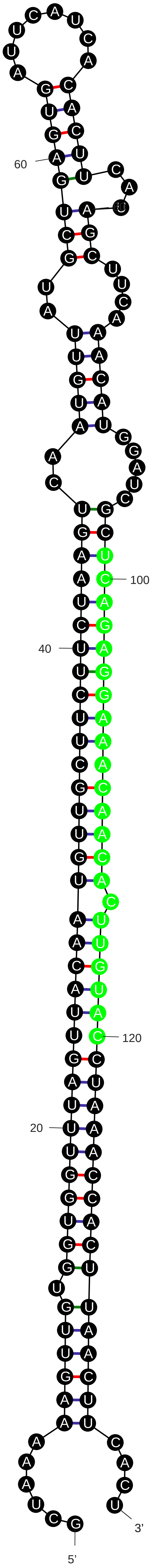

dG = -46.90 [Initially -46.90] vun_cand036

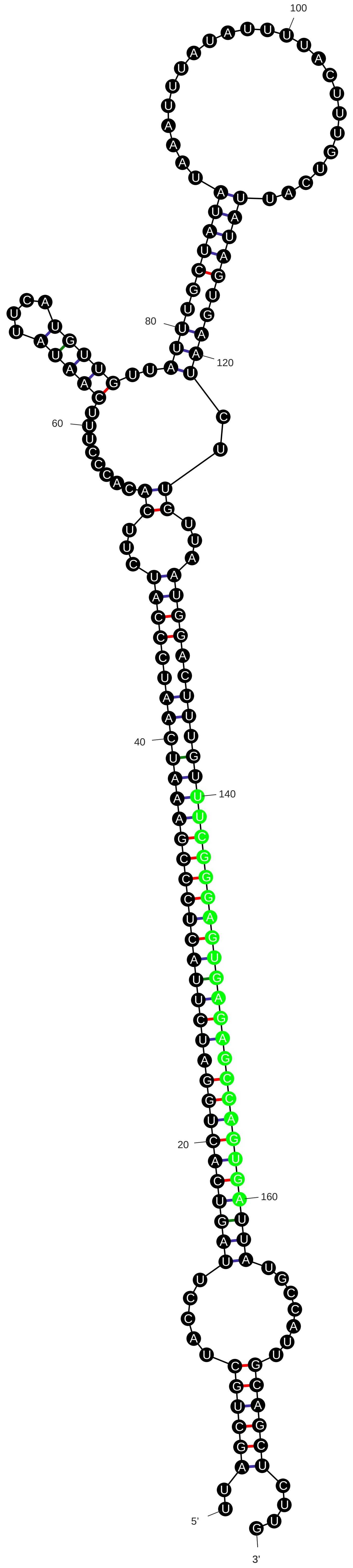

dG = -57.44 [Initially -60.30] vun_cand014
