# Supplementary material for: Identification and comparative analysis of drought-associated microRNAs in two cowpea genotypes
Source: BMC Plant Biol. 2011 Sep 17;11:127. doi: 10.1186/1471-2229-11-127 (PMC3182138; doi:10.1186/1471-2229-11-127)
Supplement: Additional file 3 — Mapping of small RNA reads from four libraries to the precursors of nine genotype-specific miRNAs. Each figure shows the precursor sequence, predicted hairpin structure, and how each unique read was mapped to the precursor. [file 1471-2229-11-127-S3.PDF]

Mapping of small RNA reads from four libraries to the precursors of nine genotype-specific miRNAs

On each map, the first line contains the miRNA family name. The second line contains the miRNA precursor sequence, with mature miRNA region in red. The third line contains the notation of secondary structure with parentheses denoting base-pairing and dots denoting mismatches or bulges. The number on the right is the free energy. Every line starting from line 4 contains the sequence, mapping position, and copy number of a mapped unique small RNA read.

```
>vun_cand058
AAGAAACGAAGACTTATTTTTAAGCAGAATGATCAAATTGTTTGCAATGACTAAATTGGGCATAATTTAATCACTGGTAACAATTTGGTCATATTGCTTAAAAATCATTGTTTTTTTAA
(((((((((.((....((((((((((..((((((((((((((..((.(((.(((((((....))))))).))).))..))))))))))))))..)))))))))).)).)))))))))...  (-39.1)
................TTTTTAAGCAGAATGATCAAA...................................................................................  1
................TTTTTAAGCAGAATGATCAAATTG................................................................................  1
.................TTTTAAGCAGAATGATCAAATTG................................................................................  1
..................TTTAAGCAGAATGATCAAATT.................................................................................  11
...................TTAAGCAGAATGATCAAATTG................................................................................  1708
...................TTAAGCAGAATGATCAAATTGT...............................................................................  2
....................TAAGCAGAATGATCAAATTG................................................................................  10
....................TAAGCAGAATGATCAAATTGT...............................................................................  865
....................TAAGCAGAATGATCAAATTGTT..............................................................................  18
.....................AAGCAGAATGATCAAATTG................................................................................  5
.....................AAGCAGAATGATCAAATTGTT..............................................................................  3
.......................GCAGAATGATCAAATTGTTTGC...........................................................................  2
........................CAGAATGATCAAATTGTTTGC...........................................................................  2
.............................................................................TAACAATTTGGTCATATTGCT......................  1
.............................................................................TAACAATTTGGTCATATTGCTT.....................  12
.............................................................................TAACAATTTGGTCATATTGCTTA....................  1
................................................................................CAATTTGGTCATATTGCTT.....................  1
................................................................................CAATTTGGTCATATTGCTTAA...................  209
................................................................................CAATTTGGTCATATTGCTTAAA..................  21
................................................................................CAATTTGGTCATATTGCTTAAAAA................  1
.................................................................................AATTTGGTCATATTGCTTAA...................  1
.................................................................................AATTTGGTCATATTGCTTAAA..................  30
.................................................................................AATTTGGTCATATTGCTTAAAA.................  2
..................................................................................ATTTGGTCATATTGCTTAAA..................  2
..................................................................................ATTTGGTCATATTGCTTAAAA.................  12
...................................................................................TTTGGTCATATTGCTTAAAA.................  1
...................................................................................TTTGGTCATATTGCTTAAAAA................  8
```

```
>vun_cand045
ACGAAGGAGGAGAAGAAAGGGAAGCTGTTCGAACTCCTTTTAGCTATGGGGCTCCAGGAAGTAAAATTCTTGTCACGGCACGTGCTGAGAAAGTTGCTTCTAACATGAGATCTAAAGTGC
..(((((((.....(((..(....)..)))...)))))))..(((.(((..(((..((((((((..(((((..((((...))))..)))))..)))))))).....)))..))).)))..  (-35.7)
............................................................................GGCACGTGCTGAGAAAGTTGCT......................  1
..............................................................................CACGTGCTGAGAAAGTTGCTT.....................  2
...............................................................................ACGTGCTGAGAAAGTTGCTTC....................  1
................................................................................CGTGCTGAGAAAGTTGCTT.....................  3
................................................................................CGTGCTGAGAAAGTTGCTTCT...................  103
................................................................................CGTGCTGAGAAAGTTGCTTCTAAC................  1
.................................................................................GTGCTGAGAAAGTTGCTTCT...................  1
....................................................................................CTGAGAAAGTTGCTTCTAACA...............  1
........................................................................................GAAAGTTGCTTCTAACATGAG...........  1
.........................................................................................AAAGTTGCTTCTAACATGAGA..........  1
```

```
>vun_cand048
TCAAAATTTTGATATATTTTGGTCTCTAAACTTTAGAAATGAATTAATATAATCATTTTAATCTAATTACGTCAATATTTTTAAAAATGTAACAAACGGTATAAACAATCTTAATACTATCAACGACATGTAAAAAAAAATTAACATAATTGAATTTAAAAAACTATATTCATTAATTTTTAAAATTTAGGAACCAAAATA
((((....))))..(((((((((..(((((.(((((((((.(((.((((((....((((((..((((((.((.(((.(((((....((((.......(((((...........))))).......))))...))))).))).)).))))))...))))))...)))))).))).))))))))).)))))..))))))))) (-35.6)
.......TTTGATATATTTTGGTCTCTA....................................................................................................................................................................... 1
.........TGATATATTTTGGTCTCTAAACT................................................................................................................................................................... 1
.............ATATTTTGGTCTCTAAACTTT................................................................................................................................................................. 1
.............ATATTTTGGTCTCTAAACTTTAGA.............................................................................................................................................................. 1
................TTTTGGTCTCTAAACTTTAGAA............................................................................................................................................................. 1
.................TTTGGTCTCTAAACTTTAGAAA............................................................................................................................................................ 59
.................TTTGGTCTCTAAACTTTAGAAATG.......................................................................................................................................................... 2
..................TTGGTCTCTAAACTTTAGAA............................................................................................................................................................. 1
..................TTGGTCTCTAAACTTTAGAAA............................................................................................................................................................ 7
..................TTGGTCTCTAAACTTTAGAAAT........................................................................................................................................................... 108
..................TTGGTCTCTAAACTTTAGAAATG.......................................................................................................................................................... 3
..................TTGGTCTCTAAACTTTAGAAATGA......................................................................................................................................................... 3
..................TTGGTCTCTAAACTTTAGAAATGAA........................................................................................................................................................ 9
...................TGGTCTCTAAACTTTAGAAAT........................................................................................................................................................... 2
...................TGGTCTCTAAACTTTAGAAATG.......................................................................................................................................................... 6
...................TGGTCTCTAAACTTTAGAAATGA......................................................................................................................................................... 2
...................TGGTCTCTAAACTTTAGAAATGAA........................................................................................................................................................ 685
...................TGGTCTCTAAACTTTAGAAATGAAT....................................................................................................................................................... 2
....................GGTCTCTAAACTTTAGAAATGAA........................................................................................................................................................ 5
....................GGTCTCTAAACTTTAGAAATGAAT....................................................................................................................................................... 4
......................TCTCTAAACTTTAGAAATGAA........................................................................................................................................................ 4
......................TCTCTAAACTTTAGAAATGAAT....................................................................................................................................................... 1
.......................CTCTAAACTTTAGAAATGAA........................................................................................................................................................ 1
........................TCTAAACTTTAGAAATGAA........................................................................................................................................................ 2
..........................TAAACTTTAGAAATGAATTAAT................................................................................................................................................... 1
.................................................................................................................................GTAAAAAAAAATTAACATAATTGAA............................................... 1
.............................................................................................................................................................................TTAATTTTTAAAATTTAGGAACC..... 1
.................................................................................................................................................................................TTTTAAAATTTAGGAACCAAAATA 2
```

>vun_cand052
GCGAACTCTCGCTTAGGCGGAGAGCTCTGGTTTTGAGCGAGGCATTGTCTCGCTCAAGCGAGAGCCACTCGCCTAAGCGAGGAGGAGTGAAAGCTTTGGG
......((((((((((((((...(((((.((((.(((((((((...))))))))))))).)))))...))))))))))))))..((((....)))).... (-56.7)
....CGAGAGCCACTCGCCTAAGCGA.................... 61
....GAGAGCCACTCGCCTAAGCGA.................... 1
....AGCCACTCGCCTAAGCGAGGAGGA.............. 1
....TCGCCTAAGCGAGGAGGAGTGA.......... 1
....TAAGCGAGGAGGAGTGAAAGCT..... 1

```
>vun_cand053
TGAATGAATATAGTCATTTTAACACAATTAAGTTAATTTTTTGTACATGTCAAACGTGTTTTATGCTATCATTTTGAGTTGTTTACACAATTTGACATGTTTCCATCTTCAATGTCTGTTGGGGAACGCATTTGACACCTCAAAAACAAATTTTAATGTAATTGAGTTAAAAGGACTATATGCATTCACTAGTGCATTAA
((((((.((((((((.(((((((.((((((..((((...(((((...((......(((((..((((...(((.(..((((((....))))))..).)))......((..((((....))))..))..))))..)))))..))...)))))..))))..)))))).))))))).)))))))).))))))............ (-55.2)
TGAATGAATATAGTCATTTTAAC................................................................................................................................................................................. 1
..AATGAATATAGTCATTTTAACA................................................................................................................................................................................ 1
...ATGAATATAGTCATTTTAACACAA............................................................................................................................................................................. 1
..............................................................ATGCTATCATTTTGAGTTGTTTAC.................................................................................................................. 1
..................................................................................................................................................CAAATTTTAATGTAATTGAGTTA............................... 1
........................................................................................................................................................TTAATGTAATTGAGTTAAAAGGAC........................ 1
.........................................................................................................................................................TAATGTAATTGAGTTAAAAGGA......................... 1
..........................................................................................................................................................AATGTAATTGAGTTAAAAGGACT....................... 1
..........................................................................................................................................................AATGTAATTGAGTTAAAAGGACTA...................... 4
...........................................................................................................................................................ATGTAATTGAGTTAAAAGGACTAT..................... 1
............................................................................................................................................................TGTAATTGAGTTAAAAGGACTA...................... 1
.............................................................................................................................................................GTAATTGAGTTAAAAGGACTA...................... 1
.............................................................................................................................................................GTAATTGAGTTAAAAGGACTAT..................... 3
.............................................................................................................................................................GTAATTGAGTTAAAAGGACTATA.................... 3
.............................................................................................................................................................GTAATTGAGTTAAAAGGACTATAT................... 34
..............................................................................................................................................................TAATTGAGTTAAAAGGACTATA.................... 11
..............................................................................................................................................................TAATTGAGTTAAAAGGACTATAT................... 5
................................................................................................................................................................ATTGAGTTAAAAGGACTATAT................... 1
```

```
>vun_cand054
TGTGCTTGTTGCGTTCCTGAAAACCTCCATCTTCAACTTGCTCATGGATAATGATTGTTGAGCAAGTTGAGGATGGAGCTTCTCCGGACGTTACGTCCTT
.......((..(((.((.((.((.((((((((((((((((((((..(((....)))..)))))))))))))))))))).)).)).)))))..))...... (-44.8)
.......................CCTCCATCTTCAACTTGCTCA........................................................ 79
........................CTCCATCTTCAACTTGCTCA........................................................ 1
..........................CCATCTTCAACTTGCTCA........................................................ 1
.........................................................TTGAGCAAGTTGAGGATGGAGCT.................... 4
.........................................................TTGAGCAAGTTGAGGATGGAGCTT................... 12
..........................................................TGAGCAAGTTGAGGATGGAGCT.................... 1
..........................................................TGAGCAAGTTGAGGATGGAGCTT................... 2
............................................................AGCAAGTTGAGGATGGAGCT.................... 49
............................................................AGCAAGTTGAGGATGGAGCTT................... 370
```

```
>vun_cand055
AAACAGAACCTTAAGCACTCCACTGTAGTAGCTCTCGCTCAAGGTAGACAATTTAGCCCAGGCGAGAGGCCATCTTGCTTAGGCGAGTTAGTCTCGCCTGAGCGAGGCTTTAACAGTGGC
...................(((((((...(((.((((((((.((((((((((((.(((.((((((((.....)))))))).)))))))).)))).))))))))))))))...))))))). (-56.2)
......AACCTTAAGCACTCCACTGTAGTA.......................................................................................... 3
............AAGCACTCCACTGTAGTAGCTC...................................................................................... 2
............AAGCACTCCACTGTAGTAGCTCTC.................................................................................... 1
.............AGCACTCCACTGTAGTAGCTCT..................................................................................... 4
...............CACTCCACTGTAGTAGCTCTC.................................................................................... 2
...............CACTCCACTGTAGTAGCTCTCG................................................................................... 2
................ACTCCACTGTAGTAGCTCTCGC.................................................................................. 2
................ACTCCACTGTAGTAGCTCTCGCTC................................................................................ 2
..................TCCACTGTAGTAGCTCTCGCTC................................................................................ 1
...................CCACTGTAGTAGCTCTCGCTC................................................................................ 3
...................CCACTGTAGTAGCTCTCGCTCA............................................................................... 48
...................CCACTGTAGTAGCTCTCGCTCAAG............................................................................. 8
....................CACTGTAGTAGCTCTCGCTCA............................................................................... 7
....................CACTGTAGTAGCTCTCGCTCAA.............................................................................. 19
.....................ACTGTAGTAGCTCTCGCTC................................................................................ 1
.....................ACTGTAGTAGCTCTCGCTCA............................................................................... 2
.....................ACTGTAGTAGCTCTCGCTCAAG............................................................................. 15
.............................................AGACAATTTAGCCCAGGCGAGA..................................................... 1
............................................................................GCTTAGGCGAGTTAGTCTCGCCTG.................... 1
..................................................................................................TGAGCGAGGCTTTAACAGTGGC 1
```

>vun_cand036

```
GCTAAAAAGTTGTGGTGGTTTAGTTACAATGTTGCTTCTTCTAAGTCAATGTTATGCTGAGTGATTCATCACACTTCATAGCTTCAAACATGGATCGCTCAGAGGAAACAACACTTGTACCTAAACCACTTAACTTCACT
......((((...((((((((((.((((((((((.(((((((.(((..(((((..((((((((........)))))...)))....))))).....))).))))))).))))).))))).))))))))))..))))....  (-45.6)
........................TACAATGTTGCTTCTTCTAA................................................................................................  1
........................TACAATGTTGCTTCTTCTAAG...............................................................................................  2
........................TACAATGTTGCTTCTTCTAAGT..............................................................................................  2
........................TACAATGTTGCTTCTTCTAAGTCA............................................................................................  1
.........................ACAATGTTGCTTCTTCTAAGT..............................................................................................  1
..........................................AAGTCAATGTTATGCTGAGTGA............................................................................  1
......................................................................ACACTTCATAGCTTCAAACATGGA..............................................  1
.......................................................................CACTTCATAGCTTCAAACATGGA..............................................  1
.......................................................................CACTTCATAGCTTCAAACATGGAT.............................................  1
..........................................................................TTCATAGCTTCAAACATGGATC............................................  1
.............................................................................ATAGCTTCAAACATGGATCGCT.........................................  1
...............................................................................AGCTTCAAACATGGATCGCTCA.......................................  1
.................................................................................CTTCAAACATGGATCGCTCAGAGG...................................  2
..................................................................................TTCAAACATGGATCGCTCAGAG....................................  1
...................................................................................TCAAACATGGATCGCTCAGAG....................................  1
.....................................................................................AAACATGGATCGCTCAGAGGAAAC...............................  3
......................................................................................AACATGGATCGCTCAGAGGAAACA..............................  1
.........................................................................................ATGGATCGCTCAGAGGAAACAACA...........................  3
..................................................................................................TCAGAGGAAACAACACTTGTAC....................  56
...................................................................................................CAGAGGAAACAACACTTGTACC...................  17
```

```
>vun_cand014
TTAGCTGCTACCTTAGTCACTGGATCTTACTCCCGAAATCAATCCCATCTTCACACCCTTTCAATATTCATGTTGTTATTTGCTATATAAATTTATATTTTACTTTGTCATTATAGTGAATCTTGTTAATGGACTTTGTTTCGGGAGTGAGAGCCAGTGATTATGCCATTGCAGCTCTTG
..((((((.....((((((((((.((((((((((((((.(((..((((...((........(((((....)))))..(((..(((((........................)))))..)))..))...))))...))))))))))))))))).)))))))))).......))))))....  (-60.7)
..........................................................................................................................................TTTCGGGAGTGAGAGCCAGTG..................... 34
...........................................................................................................................................TTCGGGAGTGAGAGCCAGTG..................... 1
...........................................................................................................................................TTCGGGAGTGAGAGCCAGTGA.................... 46
............................................................................................................................................TCGGGAGTGAGAGCCAGTG..................... 1
..............................................................................................................................................GGGAGTGAGAGCCAGTGA.................... 1
```
